# Effect of Whole Lung Lavage for Pulmonary Alveolar Proteinosis on Circulatory Dynamics: A Case Report

**DOI:** 10.7759/cureus.79354

**Published:** 2025-02-20

**Authors:** Junichi Sato, Tomohiro Chaki, Chinami Kaga, Mariko Ikeshima, Michiaki Yamakage

**Affiliations:** 1 Department of Anesthesiology, Sapporo Medical University School of Medicine, Sapporo, JPN

**Keywords:** anesthesiology, cardiac output monitoring, circulatory dynamics, pulmonary alveolar proteinosis, whole lung lavage

## Abstract

Whole lung lavage (WLL) is a standard treatment for severe pulmonary alveolar proteinosis (PAP) and is performed under general anesthesia. However, the management of anesthesia in PAP patients poses significant challenges due to their compromised respiratory function. Additionally, the instillation of large volumes of normal saline during WLL may adversely affect circulatory dynamics. In this case report, we examined the impact of WLL on circulatory parameters in a patient with severe PAP. A 71-year-old male diagnosed with idiopathic PAP underwent WLL under general anesthesia. Each lung was instilled with 1,000 mL of normal saline, followed by drainage in a series of 10 cycles per lung. Hemodynamic parameters were monitored throughout the procedure. Statistical analyses were performed to evaluate changes in circulatory values before and after saline instillation and to compare differences in hemodynamic responses between the right- and left-sided WLL. Our findings demonstrated that saline instillation during WLL significantly suppressed cardiac output. Furthermore, the suppression of circulatory function was more pronounced during right-sided WLL compared with left-sided WLL. These observations highlight the critical need for careful hemodynamic monitoring and management during WLL to ensure patient safety and optimize outcomes.

## Introduction

Pulmonary alveolar proteinosis (PAP) is a rare lung disease characterized by the accumulation of lipoproteinaceous material derived from surfactants within the alveoli, leading to impaired gas exchange and progressive respiratory insufficiency [1]. Currently, the morbidity rate of PAP in Japan is 1.65 per million [2]. Idiopathic PAP accounts for the majority of cases, with patients often presenting with progressive dyspnea, hypoxemia, and radiographic findings such as the characteristic "crazy-paving" pattern on computed tomography scans [3].

Whole lung lavage (WLL) is widely recognized as the standard treatment for patients with severe PAP [2,4]. This procedure involves the sequential instillation and drainage of large volumes of normal saline into and out of each lung under general anesthesia, effectively removing the accumulated material from the alveoli [5]. While WLL has proven to be an effective therapeutic intervention, its execution presents unique challenges, particularly due to the risks associated with anesthesia, one-lung ventilation, and hemodynamic instability [6-9]. Patients with PAP often have significant baseline respiratory compromise, necessitating careful preoperative evaluation and perioperative management to minimize the risk of hypoxemia and other complications.

The effects of WLL on respiratory function have been extensively documented, yet its impact on circulatory dynamics remains less thoroughly explored. Previous studies have suggested that this process may affect circulatory dynamics, potentially due to increased intrathoracic pressure and its effects on pulmonary and systemic circulation [10]. There are limited studies on the effects of WLL on circulatory dynamics, and those studies revealed changes in circulatory dynamics during WLL. However, the changes in circulatory dynamics between right-sided and left-sided WLL remain unclear. Additionally, it is necessary to clarify whether the problem of anesthesia is limited to oxygenation or whether circulatory control is also involved.

In this case report, we analyzed the hemodynamic changes during the WLL procedure in a patient with severe PAP and statistically compared the impact of WLL on the right lung and left lung. By focusing on these circulatory dynamics, we aim to enhance the understanding of circulatory changes associated with the WLL procedure and provide essential information for establishing safer perioperative management of patients with severe PAP.

## Case presentation

A 71-year-old male (weight: 60.3 kg, height: 158 cm) with hypertension, type 2 diabetes mellitus, and dyslipidemia had been diagnosed as having idiopathic PAP. He had a cough at rest and breathing difficulty despite oxygen administration. Percutaneous oxygen saturation (SpO_2_) was 96% (2 L/minute at rest from a nasal cannula), and 6 L/minute oxygen was needed in motion. KL-6 had increased by 23,820 U/mL, and a chest computed tomography scan showed a crazy-paving appearance spreading diffusely in both lungs. He was diagnosed with severe PAP and was scheduled for WLL under general anesthesia.

On arrival in the operating room, the baseline vital signs were heart rate (HR) 72 bpm, blood pressure (BP) 147/96 mmHg, and SpO_2_ 96% (2 L/minute, nasal cannula). General anesthesia was induced with propofol (target effect-site concentration at 3.0 µg/mL using target-controlled infusion (TCI) mode), fentanyl at 50 µg, remifentanil at 0.27 µg/kg/minute, and rocuronium at 50 mg. Anesthesia was maintained with propofol (2.7-3.0 µg/mL using TCI), remifentanil (0.03-0.14 µg/kg/minute), and intermittent administration of rocuronium (10 mg). Airway management during WLL was performed by one-lung ventilation using a 37 Fr double-lumen tube (DLT) (COOPDECH, Daiken Medical Co., Ltd., Osaka, Japan) for the left side, with a positive end-expiratory pressure (PEEP) of 4 cmH_2_O. Direct measurement of arterial pressure was monitored by placing a 22-gauge cannula in the left radial artery, and circulatory dynamics were evaluated using ProAQT^TM^ (Getinge AB, Gothenburg, Sweden), which is an arterial line system capable of continuously analyzing pulse contour.

The results of arterial blood gas (ABG) analysis just after intubation (pre-WLL) are shown in Table 1. WLL was performed in the order from the right lung to the left lung in a supine position. A lung was filled with 1,000 mL of normal saline, and lung lavage fluid was drained as much as possible. We repeated the WLL 10 times for each lung. The drainage/filling volumes of the right lung and left lung were 9,790 mL/10,000 mL and 9,330 mL/10,000 mL, respectively. There was some leakage from a junction with the DLT and a tube for instilling normal saline solution between the fourth WLL and fifth WLL for the left lung, and the procedure was temporarily interrupted to fix the WLL system. During the WLL procedure, the patient was treated with 100% oxygen, phenylephrine at 0.1 mg (administration repeated 17 times), 0.08-0.11 µg/kg/minute (approximately 160 minutes), and ephedrine at 5 mg (administration repeated six times) to prevent hypotension (mean BP < 65 mmHg). After finishing the WLL procedure, the DLT was replaced with a single-lumen tube in the operating room. On departure from the operating room (post-WLL), the vital signs were HR at 63 bpm, arterial blood pressure (ABP) at 86/64 mmHg, and SpO_2_ at 97% (FiO_2_ 1.0). The results of the ABG analysis are shown in Table 1. WLL increased pO_2_ levels and decreased pCO_2_, HCO_3_, and base excess (BE) levels. The patient was moved to the intensive care unit. He was extubated the following day (17 hours after anesthesia termination) and discharged without complications on postoperative day six.

**Table 1:** Arterial blood gas (ABG) analysis results just after intubation (pre-WLL) and upon departure from the operating room (post-WLL) The patient was continuously treated with FiO_2_ 1.0. WLL: whole lung lavage; pO_2_: partial pressure of oxygen; pCO_2_: partial pressure of carbon dioxide; HCO_3_: bicarbonate; BE: base excess

Parameters	Pre-WLL	Post-WLL	Reference Range
pH	7.286	7.297	7.350–7.450
pO_2_ (mmHg)	89.8	167.0	75.0–100.0
pCO_2_ (mmHg)	53.9	44.6	35.0–45.0
HCO_3_ (mmHg)	24.9	21.1	20.0–26.0
BE (mmol/L)	-2.1	-4.8	-3.0 to +3.0

Analysis of circulatory dynamics

**Figure 1:**
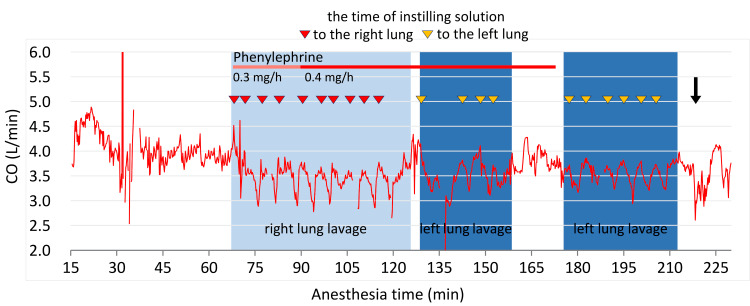
Time course of the change in cardiac output during whole lung lavage (WLL) The background color indicates WLL for the right and left lungs. The gap area in dark blue indicates the suspended procedural period due to normal saline solution leaking from the connection between the tracheal tube and the instilling tube. After the WLL procedure, a recruitment maneuver was employed (black arrow). Instilling a solution into the lungs rapidly suppresses CO in both lungs. However, the reduction in CO was more pronounced in the right lung. CO: cardiac output

A total of 10 datasets for various parameters of circulatory dynamics were obtained for each lung. The datasets included data for cardiac output (CO), cardiac index (CI), systolic arterial pressure (SAP), diastolic arterial pressure (DAP), mean arterial pressure (MAP), HR, stroke volume index (SVI), systemic vascular resistance index (SVRI), stroke volume variation (SVV), and left ventricular contractility (dPmx). Each dataset consisted of time-course changes during a single lavage, which was from before filling normal saline solution and after draining lung lavage fluid, excluding the measurement during suspended WLL (Figure 1).

The changes in circulatory parameters caused by the instillation of normal saline and the differences in hemodynamic changes between the right lung and the left lung were analyzed for each parameter. The maxima and the minima of each parameter before and after saline infusion (10 times on each side, for a total of 20 times) are compared. Statistical analyses were performed using GraphPad Prism 10 (GraphPad Software, Boston, MA). The Shapiro-Wilk test was used to assess the normality of the data distribution. A paired t-test was used to compare the hemodynamic parameters, and a p-value less than 0.05 was considered statistically significant.

The instillation of normal saline solution caused significant decreases in all measurement parameters (Table 2).

**Table 2:** Hemodynamic changes caused by the instillation of normal saline in the lung during whole lung lavage Data are presented as means ± standard deviation. The Shapiro-Wilk test was used to assess the normality of the data distribution. A paired t-test was used for statistical analysis. The paired t-test was performed to compare the values, the maxima and the minima of each parameter before (pre) and after (post) saline infusion (negative values indicate a decrease), and the p-values were calculated accordingly. CI: confidence interval; CO: cardiac output; SAP: systolic arterial pressure; DAP: diastolic arterial pressure; MAP: mean arterial pressure; HR: heart rate; SVI: stroke volume index; SVRI: systemic vascular resistance index; dPmx: left ventricular contractility

Parameters	Pre	Post	Mean Difference	95% CI	Test Statistic	P-value
CO (L/minute)	3.8±0.2	3.0±0.3	-0.79	-0.63 to -0.95	-10.2	<0.001
CI (L/minute)	2.3±0.1	1.9±0.2	-0.49	-0.39 to -0.59	-10.2	<0.001
SAP (mmHg)	104±16	77±11	-27	-20 to -34	-8.49	<0.001
DAP (mmHg)	51±6.8	40±5.5	-11	-8.7 to -14	-9.01	<0.001
MAP (mmHg)	68±10	52±7.2	-17	-12 to -21	-8.25	<0.001
HR (/minute)	71±4.0	65±4.9	-5.9	-4.3 to -7.4	-7.71	<0.001
SVI (mL/m^2^/beat)	33±1.9	27±4.0	-5.7	-4.3 to -7.1	-8.42	<0.001
SVRI (dyn·s/cm^5^/m^2^)	2378±448	1852±260	-526	-355 to -698	-6.42	<0.001
SVV (%)	13±4.4	7.6±2.4	-5.8	-4.5 to -7.2	-9.02	<0.001
dPmx (mmHg/second)	715±186	482±103	-233	-169 to -298	-7.60	<0.001

A comparison of hemodynamic data for the right and left lungs showed that HR, SVI, SVRI, and SVV were not significantly different, but the reductions of CO, CI, SAP, DAP, MAP, and dPmx in the right lung WLL were significantly larger than those in the left lung WLL (Table 3).

**Table 3:** Changes in circulatory dynamics caused by the instillation of normal solution in the right and left lung lavage Data are presented as means ± standard deviation. The Shapiro-Wilk test was used to assess the normality of the data distribution. A paired t-test was used for statistical analysis. The paired t-test was performed to compare the right lung WLL and the left lung WLL values, the difference in each parameter before and after saline infusion (negative values indicate a decrease), and the p-values were calculated accordingly. CI: confidence interval; CO: cardiac output; SAP: systolic arterial pressure; DAP: diastolic arterial pressure; MAP: mean arterial pressure; HR: heart rate; SVI: stroke volume index; SVRI: systemic vascular resistance index; dPmx: left ventricular contractility

Parameters	Right Lung	Left Lung	Mean Difference	95% CI	Test Statistic	P-value
CO (L/minute)	-0.9±0.3	-0.7±0.4	0.2	0.1 to 0.4	2.40	0.040
CI (L/minute)	-0.6±0.2	-0.4±0.2	0.1	0.0 to 0.3	2.31	0.046
SAP (mmHg)	-34±17	-20±7.0	13	2.0 to 34	2.67	0.026
DAP (mmHg)	-14±6.0	-8.3±3.3	6.0	1.4 to 11	2.92	0.017
MAP (mmHg)	-21±10	-12±4.5	8.5	0.9 to 16	2.53	0.032
HR (/minute)	-7.1±4.0	-4.6±2.2	2.5	-0.4 to 5.4	1.93	0.085
SVI (mL/m^2^/beat)	-6.4±3.1	-5.0±2.9	1.4	-1.4 to 4.2	1.15	0.281
SVRI (dyn·second/cm^5^/m^2^)	-695±354	-358±308	336	-2.2 to 675	2.25	0.051
SVV (%)	-5.2±2.9	-6.4±2.9	-1.2	-3.4 to 0.95	-1.26	0.239
dPmx (mmHg/second)	-308±153	-159±64	149	64 to 233	3.98	0.003

## Discussion

Treatment with WLL increased PaO_2_ and decreased PaCO_2_, suggesting an improvement in pulmonary exchange capacity. In this case, the hemodynamic changes were causally associated with the instillation of normal saline solution into the lung. The instillation of normal saline caused an increase in intra-alveolar pressure, and the alveolus was therefore extended. That caused the capillary area around the alveolus to be expanded and compressed flatly, and pulmonary vascular resistance (PVR) subsequently increased. Then, the elevated PVR increased right ventricular afterload and decreased left ventricular preload, which was caused by reduced pulmonary blood volume (PBV). Changes in the afterload and the preload will suppress the circulation while WLL is performed [10,11]. The circulatory suppressive effect of WLL was more significant in the right lung than in the left lung in this case. The right atrium and right ventricle, superior vena cava, and inferior vena cava are easily compressed due to their low-pressure circulatory system. Considering that the right-sided flow system has lower blood pressure and lower elasticity of blood vessels than those of the left-sided flow system and greater lung volume and blood flow in the right lung than the left lung, the exogenous pressure has a stronger effect on the right-sided flow circulation system than on the left-sided one. Therefore, hemodynamic changes during WLL are thought to be suppressed to a greater extent in the right lung than in the left lung. While the effect of the order of WLL on circulatory dynamics cannot be entirely ruled out, we believe its impact is minimal. If residual alveolar lavage fluid remains in the right lung, instilling normal saline solution in the left lung may further increase intrathoracic pressure and exert a greater influence on circulation. For example, HR would be expected to show a significant difference; however, no such difference was observed.

Patients with severe PAP need WLL but could be exposed to hypoxemia during the procedure. In the case of severe hypoxemia, standby extracorporeal membrane oxygenation (ECMO) will be often used [11-14]. There is no consensus regarding the type of ECMO, venovenous (VV)- or venoarterial (VA)-ECMO, that should be used during WLL. Considering PAP's pathological background, hypoxemia during the WLL procedure is caused by dysfunction of the alveoli and/or one-lung ventilation, and VV-ECMO may be selected for helping gas exchange because VV-ECMO has advantages over VA-ECMO such as less risk of neurologic injury and stroke [11,12,14,15]. As already stated, WLL in the right lung strongly suppresses circulation. Patients who have deterioration of cardiac function may need help with cardiocirculatory dynamics. VA-ECMO may be preferred to VV-ECMO in those cases because they need cardiopulmonary support. Therefore, evaluation of pre-procedural cardiac function will help to determine which type of ECMO is appropriate.

We experienced some leakage during the fourth and fifth WLL procedures for the left lung, and the precise drainage and filling volumes of the left lung were unknown. This may have affected the timing of extubation. Although oxygenation improved following WLL, some lavage fluid was presumed to have remained in the lungs. We anticipated that this residual fluid would be naturally absorbed or expelled. Furthermore, given the patient's advanced age and reduced physical strength, he was managed in the ICU and extubated the following morning, 17 hours after anesthesia termination. Performing lung ultrasound in the ICU to monitor temporal changes in the lungs might have facilitated earlier extubation [16-19].

In this report, only one case was presented, which is insufficient to support our conclusion. We need to examine more cases in the future, but it may be difficult to examine a sufficient number of cases because PAP is a rare disease. We did not measure and evaluate the pressure of the right heart circulation and lungs using non-invasive devices, such as echocardiography. This is one of the limitations of this article. An echocardiogram during the WLL could have helped in the evaluation. We mainly used phenylephrine, which is a pure alpha-1 adrenergic agonist, as a vasopressor, but if we had chosen other vasopressors (e.g., noradrenaline or ephedrine, which are both alpha and beta-adrenergic agonists), the results might have been different. Nevertheless, these circulation dynamics will generally apply to other PAP patients.

## Conclusions

This case highlights the significant impact of WLL on circulatory dynamics in a patient with severe PAP. Our findings demonstrate that the instillation of normal saline into the lungs suppresses hemodynamic parameters, with a more pronounced effect during right-sided WLL compared to left-sided WLL. These changes are likely attributable to increased PVR and reduced preload and afterload, particularly in the right-sided circulation system. For patients undergoing WLL, careful preoperative assessment of cardiac function and close intraoperative hemodynamic monitoring are essential to mitigate potential circulatory complications. Although this report is limited by its single-case nature, it provides valuable insights into the circulatory effects of WLL and underscores the importance of individualized anesthetic management strategies for PAP patients. Future studies are needed to validate these findings and refine clinical approaches for optimizing outcomes during WLL procedures.
